# Allergy, inflammation, hepatopathy and coagulation biomarkers in dogs with suspected anaphylaxis due to insect envenomation

**DOI:** 10.3389/fvets.2022.875339

**Published:** 2022-08-08

**Authors:** Kate Turner, Corrin Boyd, Gabriele Rossi, Claire R. Sharp, Melissa A. Claus, Abbie Francis, Lisa Smart

**Affiliations:** ^1^Emergency and Critical Care Department, School of Veterinary Medicine, Murdoch University, Perth, WA, Australia; ^2^Veterinary Pathology Department, School of Veterinary Medicine, Murdoch University, Perth, WA, Australia; ^3^Harry Butler Institute, Murdoch University, Perth, WA, Australia; ^4^Telethon Kids Cancer Centre, Telethon Kids Institute, Nedlands, WA, Australia; ^5^Discipline of Pediatrics, Medical School, The University of Western Australia, Nedlands, WA, Australia; ^6^Emergency and Critical Care Department, Small Animal Specialist Hospital, North Ryde, NSW, Australia

**Keywords:** C-reactive protein, histamine, protein C (PC), antithrombin (AT), hyaluronan, mast cell tryptase, canine anaphylaxis, cytokines

## Abstract

**Objectives:**

To compare concentrations of biomarkers of; allergy [mast cell tryptase (MCT) and histamine], inflammation [interleukin (IL)-6,-10, and−18, CXCL8, CCL2, keratinocyte chemoattractant (KC), C-reactive protein (CRP)], endothelial glycocalyx shedding (hyaluronan), coagulation [prothrombin time, activated partial thromboplastin time, fibrinogen concentration, and von Willebrand Factor antigen, protein C (PC) and antithrombin (AT) activity], and hepatopathy [alanine transaminase (ALT), aspartate transaminase (AST), alkaline phosphatase (ALP), and total bilirubin] between dogs with anaphylaxis after suspected insect exposure, dogs with critical illness, and healthy dogs.

**Design:**

This was a single center prospective clinical observational comparative biomarker study that included 25 dogs with anaphylaxis (evidence of insect exposure, acute dermatological signs, and other organ involvement), 30 dogs with other critical illness, and 20 healthy dogs. Differences across groups in biomarker concentrations were tested using one-way ANOVA or Kruskal-Wallis test, with significant *P* values (<0.05) reported for pairwise differences detected by *post-hoc* tests. Logistic regression models were used to calculate the area under the receiver operator characteristic curve (AUROC) for discrimination between anaphylaxis and non-anaphylactic illness.

**Results:**

Histamine concentration was significantly higher in the anaphylaxis group than the healthy (*P* < 0.001) and critically ill groups (*P* < 0.001), whereas no differences in MCT were detected amongst groups. Biomarker concentrations that were increased relative to healthy dogs in both the anaphylaxis and critically ill groups included IL-10 (*P* < 0.001 and *P* = 0.007, respectively), CCL2 (*P* = 0.007 and *P* < 0.001, respectively) and AST (both *P* < 0.001), whereas only the critically ill group had significantly increased CRP (*P* < 0.001), IL-6 (*P* < 0.001), KC (*P* < 0.001), ALP (*P* < 0.001), and fibrinogen (*P* = 0.016) concentrations, compared to the healthy group. Only dogs with anaphylaxis had significantly higher hyaluronan (*P* = 0.021) and ALT (*P* = 0.021) concentrations, and lower PC (*P* = 0.030) and AT (*P* = 0.032) activities, compared to healthy dogs. Both CRP and histamine concentration showed good discrimination between anaphylaxis and other critical illness, with an AUROC of 0.96 (95% CI 0.91–1) and 0.81 (95% CI 0.69–0.93), respectively.

**Conclusions:**

This preliminary study in dogs with anaphylaxis after suspected insect exposure, found evidence of an early innate immune response, glycocalyx shedding and anticoagulant consumption. Both CRP and histamine showed potential clinical utility for differentiation between anaphylaxis and other critical illness.

## Introduction

Anaphylaxis is a severe type 1 hypersensitivity reaction that causes multiple organ dysfunction, secondary to antigen exposure. Suspected insect envenomation is the most frequently reported cause in dogs (1–3). The multiple organ dysfunction is mediated by complex immunological interactions that have been characterized in mouse models and people with anaphylaxis (4, 5). The classic pathway of anaphylaxis involves prior immune cell sensitization, IgE and antigen crosslinking, and activation of mast cells and basophils. Neutrophil activation, IgG and non-immunological pathways can also contribute to the inflammatory response (4, 6, 7). These inflammatory pathways result in the release of biogenic amines, lipid mediators, cytokines, and chemokines (4, 5, 8). Anaphylaxis can also activate and modulate the coagulation system through a variety of mediators, including platelet activating factor, tryptase, chymase, and heparin (9–13). The inflammatory and coagulation pathways of anaphylaxis are presumed to be similar in dogs, however data is limited to a description of histamine and arachidonic acid pathway metabolite concentrations in an experimental model (14). Little is known on inflammatory and coagulation pathway activation during naturally-occurring anaphylaxis in dogs, though a clinical bleeding syndrome has been described in several case series (3, 15–17).

Clinical recognition of anaphylaxis relies on evidence of allergen exposure, a detail often missing from the medical history, and knowledge of the reported clinical signs, as there is no definitive diagnostic test (1, 2, 7, 17–19). Reliable diagnosis of anaphylaxis without history of allergen exposure can be difficult in both veterinary and human medicine, especially in the absence of dermatological signs (7). There are a range of candidate biomarkers that hold theoretical potential as adjunct point-of-care tests for distinguishing anaphylaxis from other critical illnesses in dogs, including biomarkers of allergy, acute inflammation and hepatopathy. Circulating allergy biomarkers, mast cell tryptase (MCT) and histamine, have been investigated for their discriminatory potential in people (8, 20–27). Despite early studies showing promise (26–28), neither biomarker has proven to be reliably increased above healthy control levels in emergency department patients with anaphylaxis (22, 23). Diagnostic utility may be hampered by variation in the magnitude of these biomarker responses in people, which may be affected by timing of sampling relative to the time course of the biomarker release, or type and route of allergen challenge. Drug and food allergy is most frequently reported in people presenting to emergency departments (8), whereas insect exposure is most frequently reported for dogs (1, 2). Blood histamine concentration increases in dogs with experimental anaphylaxis (14, 29); however, histamine has not been investigated in naturally occurring anaphylaxis, and blood MCT concentration has not been explored in either setting.

Inflammation biomarkers of the innate immune response have been explored in people with anaphylaxis but these results have not been compared to those with critical illness in order to investigate their discriminatory potential (8, 30). Inflammation biomarkers measured at time of presentation may hold value as a negative biomarkers, where the diagnosis of anaphylaxis is more likely than other critical illness if these biomarkers are *not* elevated. Non-specific inflammation biomarkers that increase in critically ill dogs include C-reactive protein (CRP), interleukins (IL)-6, IL-10, keratinocyte chemokine (KC), C–X–C motif chemokine ligand 8 (CXCL8) and C–C motif chemokine ligand 2 (CCL2), also known as monocyte chemoattractant protein-1 (31–37). These cytokines can take hours to increase in circulation following stimulation (34–40). Circulating concentrations of these inflammatory mediators have not been characterized in dogs with anaphylaxis, either to understand the underlying mechanisms of anaphylaxis or explore diagnostic utility.

The overall aim of this study was to measure biomarkers of allergy and inflammation in dogs with anaphylaxis; to develop an understanding of the mechanisms of the clinical syndrome and for identification of potential diagnostic markers. The primary objective was to compare plasma concentrations of allergy biomarkers (MCT and histamine), inflammation mediators (IL-6,−10, and−18, CXCL8, CCL2, KC, CRP) and hyaluronan (a marker of endothelial glycocalyx shedding) between dogs with evidence of anaphylaxis after suspected insect exposure, dogs with other critical illness, and healthy controls. We hypothesized that histamine and MCT concentrations would be significantly higher, and other inflammation biomarker concentrations significantly lower, in dogs with anaphylaxis compared to non-anaphylaxis critically ill dogs. Biomarkers of hepatopathy were included for comparison, given prior evidence for their discriminatory potential (2). The secondary objective was to compare coagulation biomarkers; specifically, prothrombin time (PT), activated partial thromboplastin time (APTT), fibrinogen concentration, and activities of von Willebrand Factor antigen (vWF), protein C (PC) and antithrombin (AT) between dogs with anaphylaxis, non-anaphylaxis critically ill dogs, and healthy dogs.

## Methods

The Biomarkers of ANAphylaxis associated with hymeNopterA Sting in dogs (BANANAS) study was a single center prospective clinical observational comparative biomarker study that included 25 dogs in the anaphylaxis group, 30 dogs in the critically ill group and 20 dogs in the healthy group. All dogs were client-owned with informed owner consent obtained before blood sampling. If treatment was emergent for dogs with anaphylaxis and a pre-treatment blood sample was collected as a part of routine care, informed client consent was obtained later when appropriate. The study was approved by the Murdoch University Animal Ethics Committee (approval numbers: anaphylaxis and healthy R3090/18 566, critical illness R2964/17) and was conducted in accordance with the Australian Code for the Care and Use of Animals for Scientific Purposes. The study is reported in accordance with STROBE reporting guidelines (41).

### Anaphylaxis group

**Figure 1:**
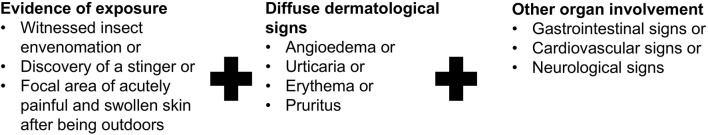
Inclusion criteria for dogs with anaphylaxis due to suspected insect exposure.

Dogs presenting between February 2019 and March 2020 were prospectively screened for inclusion criteria shown in Figure 1, which included evidence of insect exposure, acute dermatological signs, and one other organ involvement (42, 43). The mandatory inclusion of insect exposure and dermatological abnormalities was in place to increase confidence in the diagnosis of anaphylaxis. Dogs were excluded if they had a history of signs of illness for greater than 3 h, any recent non-steroidal anti-inflammatory (NSAID) or corticosteroid administration, any significant concurrent illnesses prior to anaphylaxis, if they had already received an antihistamine, corticosteroid, or exogenous catecholamine for the anaphylactic reaction, or if their body weight was <5 kg.

At the time of enrolment, treating clinicians completed a data collection sheet with information on patient signalment; dermatological, neurological and gastrointestinal signs; and cardiovascular, temperature and respiratory parameters (Supplementary Data Sheet 1). Dogs were defined as having diffuse dermatological signs if angioedema, urticaria, erythema or pruritus was present; gastrointestinal signs if vomiting, diarrhea, regurgitation, ptyalism, retching or exaggerated swallowing were present; and neurological signs if ataxia or seizures were present. Dogs were defined as having cardiovascular signs if there was presence of both an abnormal heart rate and hypotension, or either abnormal heart rate or hypotension with at least one abnormality of mucus membrane color, pulse quality or capillary refill time (CRT) (1). An abnormal heart rate was classified as bradycardia (heart rate <70 beats per minute) or tachycardia (heart rate >120 beats per minute) (44). Abnormal mucus membrane color was defined as either pale or hyperemic. Abnormal CRT was defined as <1 s or >2 s (44). Abnormal pulse quality was defined as reduced, weak or bounding (44). The data sheet also included results of diagnostic tests such as point-of-care ultrasonography, biochemistry analysis and coagulation testing, which were performed at the discretion of the treating clinician. In the case of missing data, the medical records were reviewed by K.T. prior to biomarker analysis to retrieve missing information. Blood was collected prior to any treatment, including intravenous fluid therapy, corticosteroid, antihistamine or catecholamine administration. Clinicians were encouraged to perform a focused point-of-care ultrasonogram to assess for gallbladder wall edema and abdominal free fluid during the initial treatment, and record their findings, given prior reports of its association with anaphylaxis in dogs (1, 2). Ultrasound technique was not standardized for the purposes of the study though criteria for determining gallbladder edema as previously described (1) is standard of care in the study institution. The primary clinician dictated all aspects of clinical management.

### Critical illness group

A convenience sample was used from banked samples that were stored from a randomized clinical trial of 40 dogs that required bolus intravenous fluid administration (45). Samples from that clinical trial only included those taken before the study intervention (i.e., baseline sample). Dogs were enrolled between January 2018 and February 2019. Samples were excluded if the final diagnosis was consistent with an anaphylactic reaction (*n* = 3), if there was insufficient sample (*n* = 1), or there were concerns regarding sample quality, such as lipemia (*n* = 3), delayed sample processing (*n* = 1), or incorrect labeling of aliquots (*n* = 2). The remaining 30 samples were all included. Data were collected from trial case report forms and medical records, including signalment, duration of clinical signs prior to enrolment, diagnosis, concurrent medical therapy, if any crystalloid bolus fluid therapy had been administered prior to collection of the samples and survival to hospital discharge.

### Healthy group

Healthy dogs owned by the community, staff or students were enrolled. The dogs were required to have normal physical examination, receive no current medications other than parasite prophylaxis and vaccinations, and have no known illness.

### Sample collection, processing and biomarker analysis

Six milliliters of blood was collected either from an intravenous catheter, using a three-syringe technique, or from venepuncture, and divided into ethylenediaminetetraacetic acid (EDTA), citrate and plain clot tubes. The tubes were placed on ice for no longer than 1 h until centrifugation at 1,358 g at 4°C for 10 mins. The plasma and serum were then separated into multiple aliquots. All samples were processed and frozen at −80°C within 3.5 h of collection.

Batch laboratory analysis was performed on thawed plasma or serum after storage for variable lengths of time; up to 25 months (healthy up to 5 months, anaphylaxis up to 13 months and critical illness up to 25 months) (IL-6, IL-10, IL-18, KC, CCL2, CXCL8, and coagulation parameters), up to 33 months (healthy up to 13 months, anaphylaxis up to 22 months and critical illness up to 33 months) (MCT), up to 39 months (healthy up to 17 months, anaphylaxis up to 26 months and critical illness up to 39 months) (histamine) and up to 43 months (healthy up to 19 months, anaphylaxis up to 28 months and critical illness up to 43 months) (hyaluronan, CRP, and hepatopathy parameters). All assays were performed according to manufacturer's instructions. A commercial magnetic-bead multiplex array assay (Milliplex^Ⓡ^ MAP Canine Cytokine Magnetic Bead Panel, MilliporeSigma, Burlington, MA, USA) validated by the manufacturer for canine plasma was used for measurement of IL-6, IL-10, IL-18, KC, CCL2 and CXCL8 concentration. Commercial ELISA kits were used for MCT (Dog tryptase ELISA kit, Abbexa, Cambridge, UK), histamine (Histamine ELISA kit; Beckman-Coulter, Brea, CA, USA), and hyaluronan (Quantikine Hyaluronan ELISA kit, R & D systems, Minneapolis MN, USA) validated for use in canine plasma or serum, as appropriate. All samples were run in duplicate and were repeated if the coefficient of variation was >20% (or >15% for hyaluronan), per manufacturer recommendations. Samples above the upper limit of detection were repeated at a higher dilution. Samples remaining above the upper limit of detection were assigned the upper limit of detection. Samples below the lower limit of detection but exhibiting some fluorescence were repeated without dilution. Samples remaining below the lower limit of detection were assigned the following value: Lower limit of detection/√2 (46).

A commercial turbidimetric coagulation analyser (ACL-TOP CTS 300, Instrumentation Laboratories, Bedford, MA, USA) was used to measure PT, APTT, fibrinogen concentration, and activities of AT, PC, and vWF antigen on citrated plasma. Quality assurance was performed daily on commercially available control plasmas (HaemosIL calibration plasma, Instrumentation Laboratories, Bedford, MA, USA). Pooled canine plasma was used for calibration of factor activities, and commercially available calibration plasma (HaemosIL calibration plasma, Instrumentation Laboratories, Bedford, MA, USA) was used for fibrinogen concentration. Calibration was performed for each new batch of reagent.

Batch analysis of hepatopathy biomarkers; alanine transaminase (ALT), aspartate transaminase (AST), alkaline phosphatase (ALP), and total bilirubin (Tbil) were performed using a commercial analyser (Cobas Integra 400 plus, Roche Diagnostics, Basel, Switzerland) validated for use with canine serum. Calibration and performance verification was performed using manufacturer recommended reagents (Calibrator for automated systems c.f.a.s. and PreciControl ClinChem Multi 1 and 2, Roche Diagnostics, Basel, Switzerland) prior to each use. ALT and AST were measured according to International Federation of Clinical Chemistry and Laboratory Medicine (IFCC) without pyridoxal phosphate, ALP was measured according to IFCC according to Schumann method and Tbil was measured by the colorimetric diazo method. Serum CRP was measured by particle-enhanced turbidimetric immunoassay (Canine CRP Immunoassay, Gentian AS, Moss, Norway) following 6 point calibration (Canine CRP Calibrator Kit, Gentian AS, Moss, Norway) and quality control (Canine CRP Control Kit, Gentian AS, Moss, Norway). Samples were further diluted and measurement repeated if the concentration was above the upper limit of detection.

### Statistical methods

Although this is only a preliminary biomarker study, a power calculation was performed using data from a previous biomarker study (47) to assist with the selection of sample size. This study identified that dogs with sepsis (*n* = 21) had a mean CRP concentration of 223 mg/L (SD 85) while healthy controls had a mean concentration 76 mg/L (SD 17). As we anticipated that the critical illness group would have similar concentrations to dogs with sepsis and that the anaphylaxis group would be similar to the healthy group, a sample size of 5 dogs per group was estimated to give 80% power to detect a difference between critical illness and anaphylaxis (alpha = 0.05). Given that this is a preliminary study, the anticipated variability in the groups and between different biomarkers and acknowledging that the dogs with critical illness may not be as sick as dogs with sepsis, we arbitrarily increased our sample size estimation to 30 for both the anaphylaxis and critical illness groups. A sample size of 20 was arbitrarily chosen for the healthy group, given anticipated relative homogeneity.

Statistical analysis was performed using commercial statistical software (SAS® 9.3, SAS Institute, Cary, NC). Visualization of histograms and Q–Q plots was used to assess for normal distribution. If required, log transformation of the data was performed to achieve normal distribution. Continuous data was reported as mean (95% confidence interval) when normally distributed, geometric mean (95% confidence interval) if logarithmically transformed, and median [Q1–Q3] when not normally distributed. Categorical data was reported as number (percentage). Due to the clinical relevance of measurement of PT and APTT, individual dogs with values above the error interval of the healthy control dogs were further identified.

Differences across groups were assessed using one-way ANOVA for normally distributed parameters and the Kruskal-Wallis test for non-normally distributed parameters. Where a significant difference was detected across groups, pairwise comparisons were performed with the Tukey-Kramer test for normally distributed parameters and the Dwass-Steel-Critchlow-Fligner test for non-normally distributed parameters. Univariate logistic regression models were used to calculate the area under the receiver operator characteristic curve (AUROC) for each biomarker's ability to discriminate anaphylaxis from critical illness. The AUROC and 95% confidence interval are reported. An AUROC was also calculated from a multivariate model containing all biomarkers with AUROC > 0.8 on univariate analysis. Given the preliminary nature of this study, that the comparison group was a convenience sample and there was lack of a validation cohort, sensitivity and specificity cut-points were not determined. A *P* value of <0.05 was considered significant for all tests.

## Results

### Characteristics

Two hundred and fifty-five dogs with suspected type I hypersensitivity reaction secondary to insect envenomation were screened for anaphylaxis group eligibility during the study period. Two hundred and twenty-one dogs did not meet inclusion criteria, whereby 103 dogs did not have evidence of insect exposure, 93 dogs did not have dermatological signs and 133 dogs did not have evidence of other organ involvement (i.e., dermatological only). Many of these dogs lacked multiple inclusion criteria. Thirty-four dogs met the inclusion criteria of which 9 were then excluded, leaving 25 dogs remaining in the study. Reasons for exclusion included; prior treatment (*n* = 6), body weight <5 kg (*n* = 2) and receiving lokivetmab (*n* = 1). Recruitment was discontinued at a sample size of 25 dogs due to the impact of COVID-19 restrictions at the study institution.

The most frequent breeds, and their crosses, in the anaphylaxis group were Poodles (*n* = 4; 16%), and 2 dogs each (8%) of Dachshund, Australian Kelpie, Labrador Retriever and Golden Retriever. All other breeds were only represented by an individual dog. The median weight was 10.6 kg (7.3–16.9) and median age was 1.1 years (0.4–5.2). There were 7 (28%) male neutered dogs and 6 dogs each (24%) of female spayed, female entire and male entire. Thirteen dogs (52%) had an insect stinger removed, 11 dogs had focal skin signs without a stinger found (44%) and 5 dogs were reported by the owner to have contact with a bee or ant (20%). Multiple dogs fulfilled more than one of the insect exposure criteria. Median duration of clinical signs (*n* = 19) was 1 h (0.5–1) prior to enrolment. In addition to dermatological signs, 22 dogs (88%) had gastrointestinal signs, and 21 dogs (84%) had cardiovascular signs including 6 dogs (24%) with arterial hypotension. No dogs had neurological signs reported. Additional clinical signs included increased respiratory rate or effort (*n* = 6; 24%) and increased rectal temperature (*n* = 1; 4%). Twenty-two dogs (88%) had results reported of a point-of-care abdominal ultrasound performed by the treating clinician. Of these, 13 dogs (59%) had gallbladder edema detected and 3 dogs (14%) had peritoneal effusion present. No dogs with anaphylaxis died during hospitalization; however, one dog was euthanized after a second anaphylaxis episode of marked severity, 3 months after study enrolment.

Frequent breeds, and their crosses, in the critical illness group included three dogs each (10%) of Labrador Retriever, Rottweiler, Poodle and Shih Tzu and two dogs each (6.7%) of Golden Retriever and Silky Terrier. All other breeds were only represented by an individual dog. The median weight was 25.4 kg (12.2–34.5) and median age was 7.9 years (3.8–10.5). There were 11 (37%) male neutered, 9 (30%) female spayed, 7 (23%) male entire and 3 (10%) female entire dogs. The median duration of clinical signs prior to presentation was 24 h (8–48). The most frequent diagnoses were acute hemorrhagic diarrhea syndrome (*n* = 10), gastric dilatation volvulus (*n* = 4), gastrointestinal foreign body obstruction (*n* = 3) and bite trauma (*n* = 2). Other diagnoses (1 dog each) included bicavitary effusion and pyothorax, cycad toxicosis, immune mediated polyarthropathy, hemoperitoneum with abdominal mass, mesenteric volvulus, septic peritonitis, prostatic disease, right atrial thrombosis and adrenal mass, tetanus with pneumonia, and motor vehicle accident trauma. One dog was receiving low-dose corticosteroids at the time of enrolment (<0.5 mg/kg of prednisolone every other day). Otherwise, no dogs received NSAIDs, antihistamines or other immune modulatory drugs prior to sample collection. Ten dogs received bolus administration of crystalloid fluid therapy prior to enrolment, with a median volume of 15 ml/kg (range 10–44 ml/kg). Of the dogs with critical illness, 20 (66.7%) survived to discharge, 2 (6.7%) died and 8 (26.7%) were euthanized.

The healthy group included a range of breeds, and their crosses, including Australian Shepherds (*n* = 3; 15%), and 2 dogs each (10%) of Gordon Setter, Border Collie and Kelpie. All other breeds were only represented by an individual dog. The median weight was 27 kg (16.1–33) and median age was 5.5 years (3.05–7.15). There were 9 male neutered (45%), 7 female spayed (35%), and 2 each (10%) of female entire and male entire dogs.

### Comparison of biomarkers between groups

**Figure 2:**
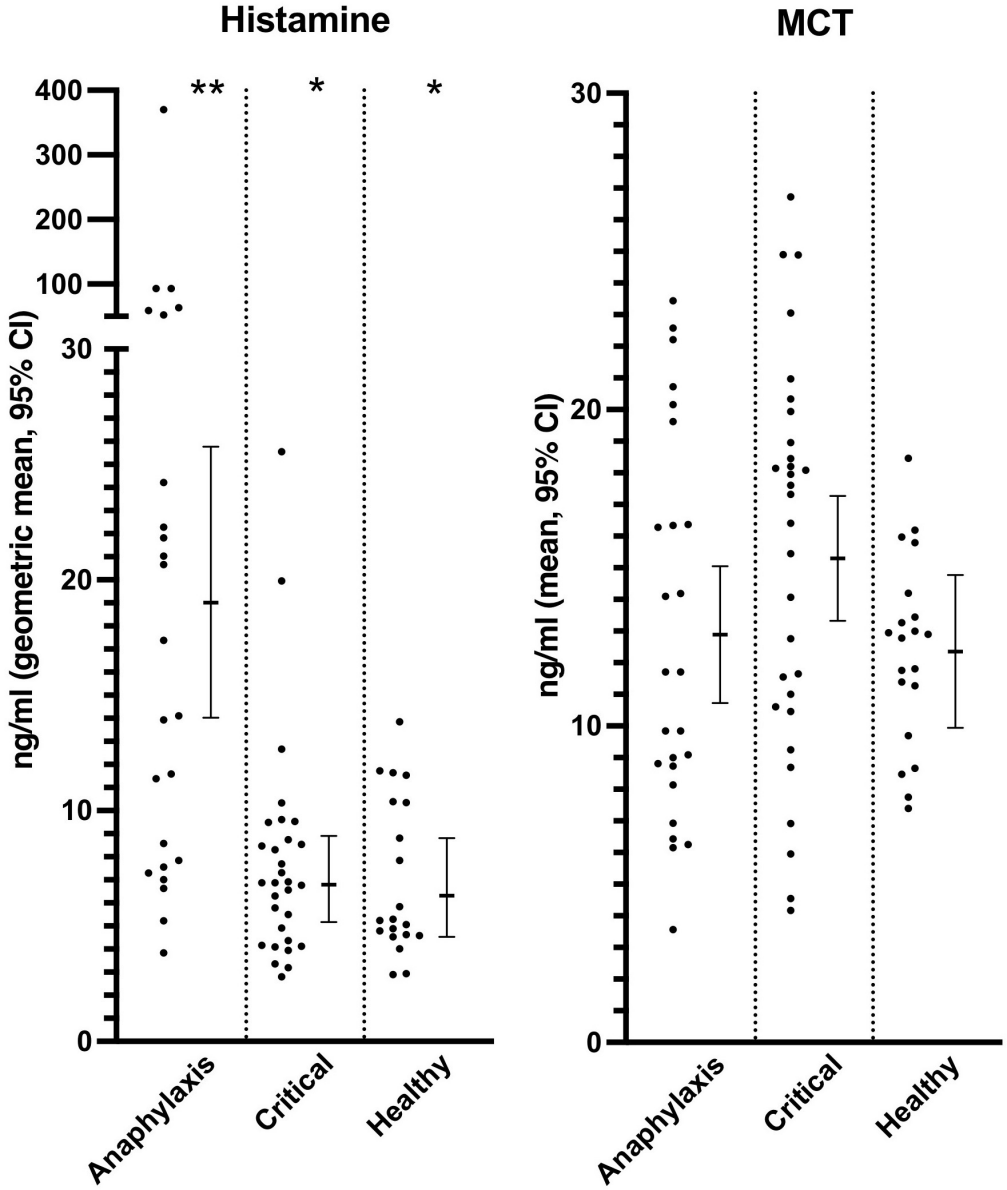
Scatter and box-and-whisker plots of serum histamine and plasma mast cell tryptase (MCT) concentrations in dogs with anaphylaxis, dogs with other critical illness and healthy dogs. The box-and-whisker plots display either mean and 95% confidence interval (MCT) or geometric mean and 95% confidence interval (histamine) as appropriate for the data distribution. * and **indicate a significant (*P* < 0.05) difference between groups.

**Figure 3:**
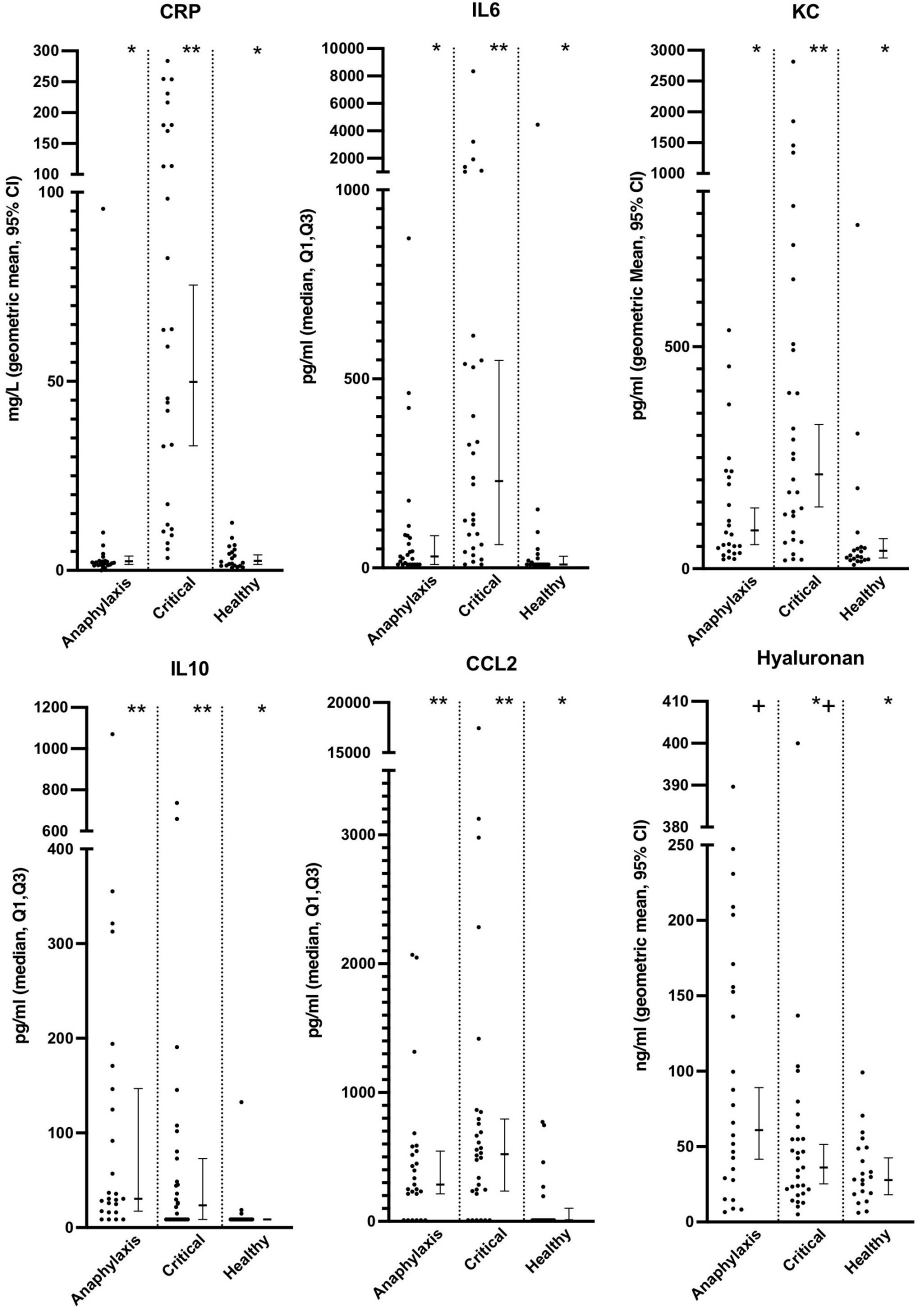
Scatter and box-and-whisker plots of C reactive protein (CRP), interleukin (IL) 6 and 10, C-C motif chemokine ligand-2 (CCL2), keratinocyte-derived chemokine (KC) and hyaluronan concentrations in dogs with anaphylaxis, dogs with other critical illness and healthy dogs. The box-and-whisker plots display either geometric mean and 95% confidence interval (CRP, KC and hyaluronan) or median and Q1, Q3 (IL6, IL10 and CCL2) as appropriate for the data distribution. * and **indicate a significant (*P* < 0.05) difference between groups.

Serum histamine concentration was significantly higher in the anaphylaxis group, compared to the critical illness group (*P* < 0.0001) and the healthy group (*P* < 0.0001) (Figure 2; Supplementary Table 1), whereas no significant differences were found in MCT between groups (*P* = 0.12) (Figure 2; Supplementary Table 1). Histamine concentration could not be measured in one dog in the anaphylaxis group due to insufficient sample volume. C-reactive protein, IL6, and KC concentrations were significantly higher in the critical illness group, compared to the anaphylaxis (CRP *P* < 0.0001, IL6 *P* < 0.001, KC *P* = 0.015) and healthy groups (CRP *P* < 0.0001, IL6 *P* < 0.0001, KC *P* < 0.0001) (Figure 3; Supplementary Table 1). In contrast, the anaphylaxis group did not have significantly higher concentrations of these three biomarkers, compared to the healthy group (CRP *P* = 0.99, IL6 *P* = 0.17, KC *P* = 0.08). C-reactive protein concentration could not be measured in two dogs in the critical illness group due to insufficient sample volume. Both IL-10 and CCL2 concentrations were significantly higher in the anaphylaxis group (IL10 *P* < 0.0001, CCL2 *P* = 0.007) and the critical illness group (IL10 *P* = 0.007, CCL2 *P* < 0.001), compared to the healthy group (Figure 3; Supplementary Table 1). There were no significant differences in CXCL8 or IL18 across groups (CXCL8 *P* = 0.067, IL18 *P* = 0.58) (Supplementary Table 1). The anaphylaxis group had a significantly higher serum hyaluronan concentration, compared to the healthy group (*P* = 0.021) (Figure 3, Supplementary Table 1). No other between-group differences in hyaluronan concentration were detected. Hyaluronan concentration could not be measured in one dog in the critical illness group due to insufficient sample volume.

**Figure 4:**
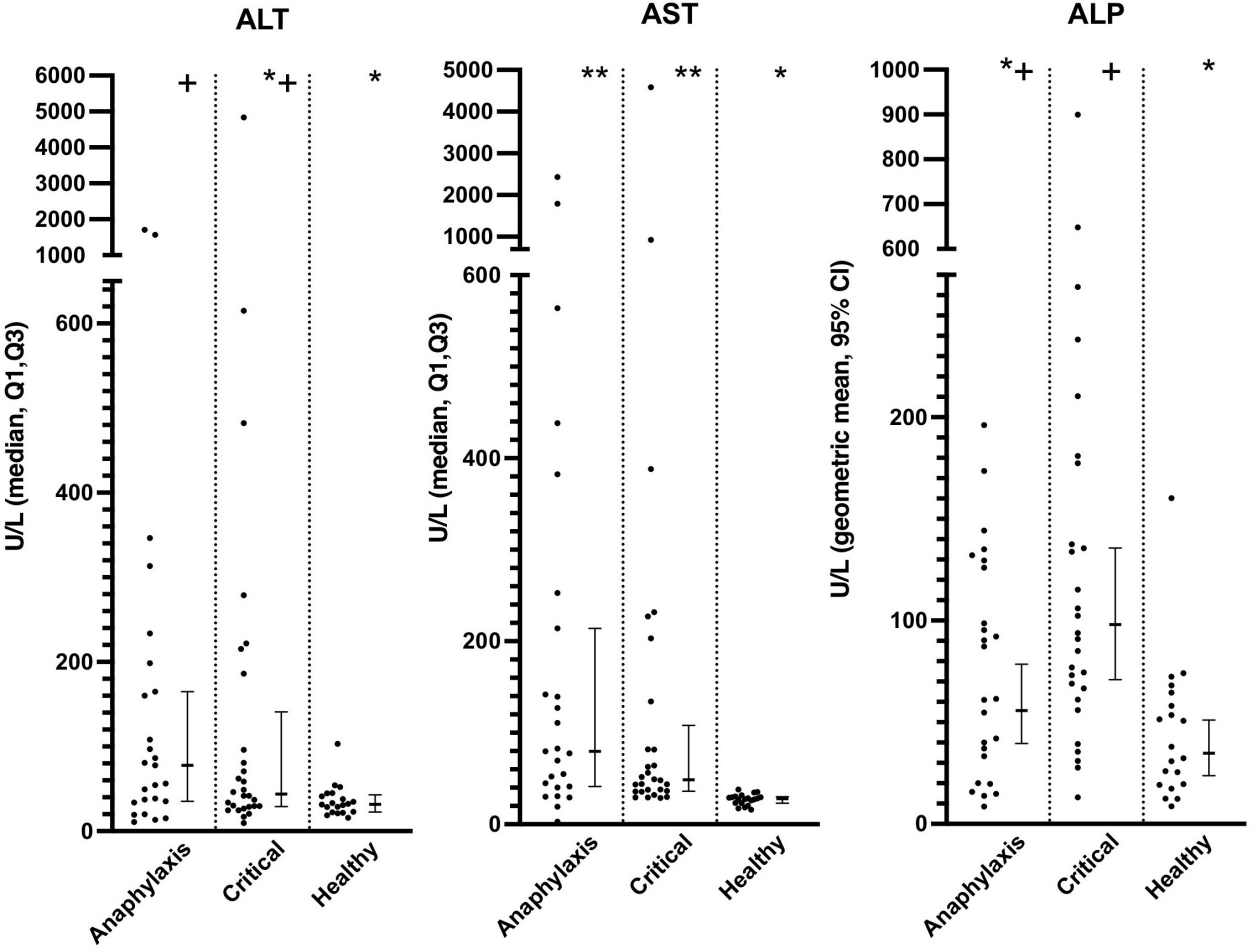
Scatter and box-and-whisker plots of alanine aminotransferase (ALT), aspartate aminotransferase (AST), and alkaline phosphatase (ALP) concentrations in dogs with anaphylaxis, dogs with other critical illness and healthy dogs. The box-and-whisker plots display either geometric mean and 95% confidence interval (ALP) or median and Q1, Q3 (ALT and AST) as appropriate for the data distribution. * and **indicate a significant (*P* < 0.05) difference between groups.

Serum AST concentrations were significantly higher in both the anaphylaxis and critical illness group, compared to the healthy group (both *P* < 0.0001), whereas only the anaphylaxis group had significantly higher ALT concentration, compared to the healthy (*P* = 0.021) group (Figure 4; Supplementary Table 1). There was no significant difference in ALT (*P* = 0.73) or AST (*P* = 0.44) concentrations between the anaphylaxis and critical illness group. Serum ALP concentration was significantly higher in the critical illness group, compared to the healthy group (*P* < 0.001), but this was the only between-group difference detected (Figure 4; Supplementary Table 1). There was no significant difference in serum Tbil concentration across groups (*P* = 0.066; Supplementary Table 1). Concentrations of AST, ALP, and Tbil could not be measured in two dogs in the critical illness group due to insufficient sample volume.

**Figure 5:**
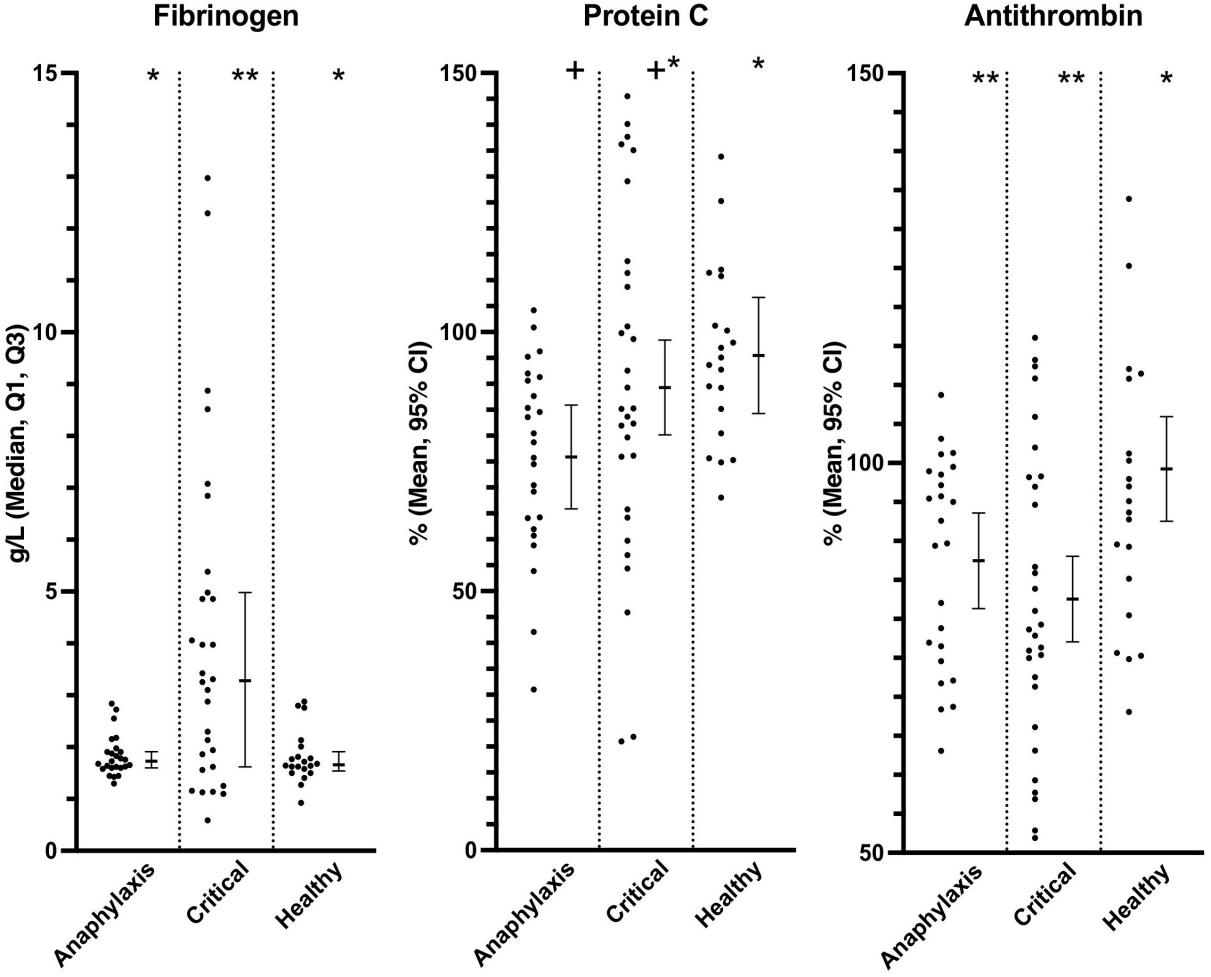
Scatter and box-and-whisker plots of fibrinogen concentration, protein C and, antithrombin activity in dogs with anaphylaxis, dogs with other critical illness and healthy dogs. The box-and-whisker plots display either mean and 95% confidence interval (protein C and antithrombin) or median and Q1, Q3 (fibrinogen) as indicated by the individual Y axis as appropriate for the data distribution. * and **indicate a significant (*P* < 0.05) difference between groups.

Plasma fibrinogen concentration was significantly higher in the critical illness group, compared to both the healthy (*P* = 0.016) and anaphylaxis (*P* = 0.016) groups; however, there was no significant difference between the anaphylaxis and healthy group (*P* = 0.89) (Figure 5; Supplementary Table 1). Plasma AT activity was significantly lower in both the anaphylaxis (*P* = 0.032) and critical illness group (*P* < 0.001), compared to the healthy group (Figure 5; Supplementary Table 1). Protein C was significantly lower in the anaphylaxis group, compared to the healthy group (*P* = 0.03); however, no other between group differences were detected (Figure 5; Supplementary Table 1). There were no significant differences across groups in PT (*P* = 0.08), APTT (*P* = 0.058) or vWF (*P* = 0.58) (Supplementary Table 1). Eight dogs from the anaphylaxis group and 15 dogs in the critical illness group had a PT greater than the 95% confidence interval of the healthy group. Thirteen dogs in the anaphylaxis group and 9 dogs in the critical illness group had an APTT greater than the Q3 of the healthy dogs.

Summarized data for all biomarkers and results of between-group statistical comparisons can be found in Supplementary Table 1. Summarized data for the critical illness group and the critical illness group excluding patients that had received prior bolus intravenous fluid therapy are included in Supplementary Table 2.

### Discriminatory potential: Anaphylaxis compared to critical illness

The AUROC and 95% confidence interval for all biomarkers are provided in Table 1, in the order of best discrimination to worst. C-reactive protein and histamine both had an AUROC >0.8; whereby dogs with anaphylaxis tended to have high histamine concentration and low CRP concentration, with the opposite occurring in dogs with critical illness. When CRP and histamine were combined in a multivariate model, the AUROC indicated near perfect discrimination at 0.96 (95% CI 0.91–1), though comparable to the CRP univariate model.

**Table 1:** Area under the receiver operator characteristic curve (AUROC) and 95% confidence interval for all biomarkers comparing dogs with anaphylaxis to those with other critical illness, in order of highest to lowest AUROC.

**Biomarker**	**AUROC**	**95% CI**
CRP	0.96	0.91–1.00
Histamine	0.81	0.69–0.93
IL-6	0.79	0.66–0.91
Fibrinogen	0.72	0.57–0.87
KC	0.70	0.56–0.84
Hyaluronan	0.65	0.50–0.81
ALP	0.65	0.50–0.80
IL-10	0.63	0.48–0.78
Protein C	0.63	0.48–0.78
CCL2	0.62	0.47–0.77
Mast cell tryptase	0.61	0.46–0.77
Bilirubin	0.60	0.45–0.76
AST	0.60	0.44–0.76
Antithrombin	0.58	0.43–0.74
IL-18	0.58	0.44–0.73
vWF	0.56	0.41–0.72
CXCL8	0.56	0.40–0.71
PT	0.56	0.40–0.71
APTT	0.55	0.38–0.71
ALT	0.44	0.28–0.60

Shaded areas indicate those biomarkers where the error interval included values below 0.5, consistent with no discrimination between groups. CRP, C reactive protein; IL, interleukin; CCL2, C-C motif chemokine ligand-2; CXCL8, C-X-C motif chemokine ligand 8; KC, keratinocyte-derived chemokine; vWF, von Willebrand factor; AST, aspartate aminotransferase; ALT, alanine aminotransferase; ALP, alkaline phosphatase; PT, prothrombin time; APTT, activated partial thromboplastin time.

## Discussion

This study detected numerous differences in allergic, inflammation, coagulation, and hepatopathy biomarkers between dogs with anaphylaxis, healthy dogs and dogs with a non-anaphylactic critical illness. These results identified that CRP alone, or the combination of CRP and histamine, may have utility as diagnostic biomarkers to differentiate anaphylaxis from non-anaphylactic critical illness in dogs.

C-reactive protein is an acute phase protein produced primarily by the liver in response to stimulation from proinflammatory cytokines, especially IL-1, IL-6 and tumor necrosis factor-α (TNF-α) (48). The lack of an increase in most individuals with anaphylaxis, compared to healthy dogs, is considered most likely due to the fast onset of clinical signs. C-reactive protein likely takes at least 4 h to increase in circulation in dogs after an inflammatory stimulus, according to experimental models (34–37, 48), whereas dogs with suspected anaphylaxis tend to present within 1–2 h of clinical signs (1). Timing of blood sampling may be the crucial aspect for identifying negative biomarkers for anaphylaxis, as suggested by this study. For example, many dogs with anaphylaxis showed increased plasma IL-10 concentration, known to increase within an hour of shock (38) or endotoxaemia (40), and some dogs showed elevated plasma IL-6 or CCL2, known to increase within 1–3 h of shock (38), endotoxaemia (40) or surgical stimulus (34, 49). This variability reduced the discriminatory potential for these inflammatory biomarkers.

In addition to CRP, plasma histamine concentration showed good discriminatory potential between dogs with anaphylaxis and non-anaphylactic critical illness, based on an AUROC of 0.81. Though over half of dogs with anaphylaxis showed plasma histamine concentration above those of healthy controls, not all did, and some dogs with critical illness also showed mild elevations. In human medicine, histamine has good specificity (0.88) but poor sensitivity (0.60) for separating anaphylaxis from healthy individuals (20), meaning a high rate of false negatives. This may be due to the time course of histamine release in people; serum histamine concentrations peak within 5–10 mins of bee envenomation challenge before returning to baseline by 30–40 mins (26). Although the time course of histamine release in people seems comparable to that of dogs in an experimental anaphylaxis model (14), the results of this study suggest that increased serum concentrations of histamine may persist longer in dogs with naturally occurring anaphylaxis, given that the median time of clinical signs prior to presentation was 1 h. Persistence in histamine elevation may also be associated with insect envenomation specifically, as this study did not include other triggers of anaphylaxis.

Given the rapid rise and decline of circulating histamine observed in people with anaphylaxis, MCT has been suggested as a superior diagnostic biomarker, as its rise and decline are slower (26). When people presenting to an emergency department with anaphylaxis were compared to those with critical illness, however, utility mimicked that of histamine concentration, with a good specificity (0.93) but poor sensitivity (0.58) (22). Interestingly, we found no differences in MCT amongst groups in this study. This may be due to a true absence of a difference between groups or may be due to failure of the ELISA utilized to detect a difference. We did not independently validate the performance of the MCT ELISA used in this study. Further research using independently validated MCT ELISA kits is warranted.

This study identified significantly higher concentrations of inflammation and endothelial biomarkers in dogs with anaphylaxis, compared to healthy dogs, including CCL2, IL-10 and hyaluronan, that have not been previously described. Further, some individual dogs had plasma IL-6 or KC concentrations higher than the 95% confidence interval of the healthy dogs. Elevations in these proinflammatory biomarkers are consistent with an early innate immune response, similar to patterns seen in biomarker studies of dogs with sepsis and other types of critical illness (40, 50). Hyaluronan is a biomarker of endothelial glycocalyx shedding and its increase suggests endothelial activation and structural alteration (38, 51). Fluid therapy can also increase hyaluronan concentration, however samples from the anaphylaxis group were taken before any treatment was given (38, 51). Loss of the endothelial glycocalyx may contribute to increased vascular permeability and play a role in fluid extravasation, which is known to occur in anaphylaxis (52). In contrast, we did not identify differences in IL-18 amongst the study groups. Interleukin-18 is a proinflammatory biomarker produced by macrophages and is increased in other types of hypersensitivity reactions in people (53, 54), therefore it was included in this study for its potential as a discriminatory biomarker. This cytokine has not been shown to be reliably increased in dogs with early critical illness (40, 50, 55, 56), which is consistent with the findings in this study, including in dogs with anaphylaxis.

Serum ALT concentration had potential as a diagnostic biomarker of anaphylaxis as it has been shown to be significantly higher in dogs with anaphylaxis, compared to dogs with a mild allergic reaction (2). This finding may be due to changes in hepatic perfusion that can occur during anaphylaxis, including obstruction of hepatic venous outflow and portal venous congestion^55−58^. This study corroborated increases in not only serum ALT but also AST concentration. Increases in AST also arise due to muscle damage, which has been reported in people following mass bee envenomation (57). However, non-anaphylactic critical illness can be associated with altered hepatic perfusion and myocyte damage, and neither of these biomarkers were found to be sufficiently discriminatory between anaphylaxis and critical illness.

Prolonged coagulation times and hemoperitoneum have been described in dogs with anaphylaxis (1, 15–17, 58), though the current mechanisms are unknown. Proposed mechanisms have included the release of heparin from mast cells, hyperfibrinolysis from tryptase production, and direct toxic effects of the Hymenoptera venom through the activity of phospholipase A_2_ (59, 60). Although we found no significant difference in mean coagulation times between dogs with anaphylaxis and healthy dogs in this study, we identified many dogs with elevation of PT and/or APTT above the error interval of healthy control times. Importantly, these changes were detected before administration of any fluid therapy. Further, concentrations of the anticoagulants measured, protein C and antithrombin, were significantly lower in dogs with anaphylaxis, compared to healthy dogs. This provides evidence that a consumptive coagulation process may be occurring. Further investigation of individual coagulation factors is required in a larger cohort of dogs with anaphylaxis, including those with clinical evidence of coagulopathy or hemoperitoneum secondary to anaphylaxis, in order to further elucidate the underlying mechanisms of disturbed coagulation.

This preliminary biomarker study has several important limitations. Firstly, the clinical criteria for identifying cases of anaphylaxis were strict compared to prior studies, requiring both dermatological signs and evidence of insect exposure (1, 2, 15, 17). Although these criteria maximize the likelihood that all dogs enrolled were truly suffering an anaphylactic reaction, it may have created bias in our study sample, reducing generalizability to all dogs with anaphylaxis. The presence of dermatological signs may have also skewed biomarker responses (21). Further, despite the strict inclusion criteria, this study may have included cases that were misdiagnosed with anaphylaxis, even though no alternate diagnosis was identified during hospitalization. The second limitation is use of a convenience sample for the critically ill group with a range of illness severity, that may not represent the type of case for which anaphylaxis is a genuine differential diagnosis. The critical illness group included dogs that had clinical signs of illness for greater than 12 h before presentation, which may have skewed results of the AUROC analysis, given timing of blood sampling is likely important for presence or absence of certain biomarker elevations. Another important consideration for the critically ill group was the administration of intravenous fluid therapy prior to sample collection in 10 dogs. This is important as large-volume bolus fluid administration may alter levels of hyaluronan, and inflammatory markers such as IL-6, 10, CXCL8 and KC and therefore may have affected these results (38, 51, 61). The true effects of intravenous fluid therapy on hyaluronan and other inflammatory biomarkers has not been fully resolved, especially when fluid therapy is utilized for the correction of hypovolaemia (51). Two studies in dogs have identified increases in hyaluronan concentration following fluid therapy for the management of either shock (38) or surgery (61), compared to baseline. However, it is difficult to separate the effect of the insult (either shock or surgery) from the effect of fluid therapy on hyaluronan shedding, and these studies may reflect a combination of both effects. A study in sheep with sepsis identified significantly higher IL-8 concentration at a singular time point (12 h) in sheep that were fluid resuscitated, compared to sheep with no fluid resuscitation, while there was no significant difference detected in hyaluronan, IL6 and IL10 concentration between groups (62). There is also a consideration of haemodilution reducing the concentration of hyaluronan and other inflammatory markers after a fluid bolus (51). The dogs that received fluid in the critical illness group before research blood sampling were not excluded from analysis due to concerns of biasing the results, however *post-hoc* summarized biomarker concentrations have been provided in Supplementary Table 2. The results of this study need to be validated in a larger cohort that includes a comparison group of acute critical illness that has clinical signs compatible with a differential diagnosis of anaphylaxis, and are enrolled prior to any rapid fluid administration. Finally, the small sample size may have hampered the ability to find significant differences amongst treatment groups for some biomarkers.

In conclusion, this study detected some important differences between allergic, inflammation, coagulation, and hepatopathy biomarkers in dogs with anaphylaxis secondary to suspected insect envenomation, healthy dogs and dogs with non-anaphylactic critical illness. We identified evidence of histamine release, an early innate immune response, endothelial glycocalyx shedding and consumption of anticoagulants in dogs with anaphylaxis prior to any treatment, relative to healthy dogs. This study also identified potential utility of serum CRP concentration, or a combination of serum histamine and CRP concentration, to distinguish anaphylaxis from non-anaphylactic illness. This result is particularly compelling given that CRP is readily available in the clinical setting, however, this result needs to be validated in a larger study.

## Data availability statement

The raw data supporting the conclusions of this article will be made available by the authors, without undue reservation.

## Ethics statement

The animal study was reviewed and approved by Murdoch University Animal Ethics Committee. Written informed consent was obtained from the owners for the participation of their animals in this study.

## Author contributions

KT contributed to study design, sample collection, laboratory analysis, and wrote the manuscript. CB and LS contributed to study design, sample collection, laboratory analysis, and revision of the manuscript. GR contributed to study design, laboratory analysis, and revision of the manuscript. MC and CS contributed to study design, sample collection, and revision of the manuscript. AF contributed to study design and revision of the manuscript. All authors contributed to the article and approved the submitted version.

## Funding

This work was supported by the Australian Companion Animal Health Fund and Caring for Pets Fund, Murdoch University.

## Conflict of interest

The authors declare that the research was conducted in the absence of any commercial or financial relationships that could be construed as a potential conflict of interest.

## Publisher's note

All claims expressed in this article are solely those of the authors and do not necessarily represent those of their affiliated organizations, or those of the publisher, the editors and the reviewers. Any product that may be evaluated in this article, or claim that may be made by its manufacturer, is not guaranteed or endorsed by the publisher.
