# Supplementary material for: Allergy, inflammation, hepatopathy and coagulation biomarkers in dogs with suspected anaphylaxis due to insect envenomation
Source: Front Vet Sci. 2022 Aug 8;9:875339. doi: 10.3389/fvets.2022.875339 (PMC9393546; doi:10.3389/fvets.2022.875339)
Supplement: Supplementary file 1 [file Data_Sheet_1.PDF]

Supplement Table 1.

Table. Comparison of biomarkers in healthy dogs,, dogs suffering an anaphylactic reaction and dogs with non-anaphylactic critical illness. Biomarkers are given as mean (95%CI), geometric mean (95% CI) or median [Q1-Q3]. Sample size for healthy dogs 20 for all biomarkers, anaphylaxis 25 for all except histamine with sample size 24, the critical illness sample size was 30 for all biomarkers except AST, ALT, ALP, bilirubin, CRP and hyaluronan which each had a sample size of 28. P values for difference between individual groups if a significant difference was detected on between group analysis. Abbreviations: CRP, C reactive protein; IL, interleukin; CCL2, C-C Motif Chemokine Ligand-2; CXCL8, C-X-C Motif Chemokine Ligand 8; KC, Keratinocyte-derived chemokine; PC, activated protein C; vWF, von Willebrand factor; PT, prothrombin time; APTT, Activated partial thromboplastin time; AT, Antithrombin; AST, Aspartate aminotransferase; ALT, Alanine aminotransferase; ALP, Alkaline phosphatase; MCT, Mast cell tryptase.

Analyte	Healthy	Critical illness	Anaphylaxis	P value			
				Across groups	Anaphylaxis vs Healthy	Anaphylaxis vs Critical illness	Critical illness vs Healthy
**Histamine (ng/mL)**	6.3 (4.5-8.8)[a]	6.8 (5.2-8.9)[a]	19.0 (14.0-25.8)[b]	<0.0001	<0.0001	<0.0001	0.94
**MCT (ng/mL)**	12.4 (9.9-14.8)	15.3 (13.3-17.3)	12.9 (10.7-15.1)	0.12	N/A	N/A	N/A
**IL6 (pg/mL)**	8.6 [8.6-30.8] [a]	229.5 [61.5-548.6] [b]	30.2 [8.6-83.4] [a]	<0.0001	0.17	0.0008	<0.0001
**CXCL8 (pg/mL)**	615 (301-1255)	587 (328-1051)	421 (222-797)	0.067	N/A	N/A	N/A
**KC (pg/mL)**	40.3 (24.0-67.7) [a]	212.6 (139.1-324.7) [b]	86.1 (54.1-137.0) [a]	<0.0001	0.08	0.015	<0.0001
**IL10 (pg/mL)**	8.6 [8.6-8.6] [a]	23.5 [8.6-73.0] [b]	30.4 [17.4-146.9] [b]	<0.0001	<0.0001	0.21	0.007
**IL18 (pg/mL)**	19.9 [8.6-61.6]	23.3 [8.6-276.6]	8.6 [8.6-85.9]	0.58	N/A	N/A	N/A
**CCL2 (pg/mL)**	8.6 [8.6-545.2] [a]	520.8 [234.6-794.9] [b]	285.5 [213.8-545.2] [b]	0.0003	0.0068	0.29	0.0004
**CRP (mg/L)**	2.53 (1.55-4.13) [a]	49.86 (32.95-75.44) [b]	2.42 (1.55-3.78) [a]	<0.0001	0.99	<0.0001	<0.0001
**Hyaluronan (ng/mL)**	27.8 (18.1-42.5) [a]	36.1 (25.4-51.4) [ab]	60.9 (41.6-89.1) [b]	0.022	0.021	0.12	0.61
**AST (U/L)**	27.9 [23.2-27.9][a]	48.6 [36.2-108.1][b]	79.8 [41.1-214][b]	<0.0001	<0.0001	0.4357	<0.0001
**ALT (U/L)**	31.9 [22.7-42.9][a]	44.0 [29.2-141.1][ab]	77.7 [35.2-165][b]	0.023	0.021	0.73	0.11
**ALP (U/L)**	34.8 (23.7-51.1)[a]	98.1 (70.9-135.7)[b]	55.7 (39.5-78.6)[ab]	0.0004	0.17	0.051	0.0003
**Bilirubin (umol/L)**	0.70 (0.44-1.13)	1.46 (0.97-2.22)	1.23 (0.77-1.98)	0.066	N/A	N/A	N/A
**Fibrinogen (g/L)**	1.66 [1.54-1.91] [a]	3.28 [1.62-4.98] [b]	1.73 [1.6-1.91] [a]	0.0045	0.89	0.016	0.016
**AT (%)**	99.2 (92.5-106.0) [a]	82.5 (77.1-88.0) [b]	87.5 (81.3-93.6) [b]	0.0011	0.032	0.46	0.0008
**PC (%)**	95.5 (84.3-106.7) [a]	89.3 (80.2-98.4) [ab]	75.9 (65.9-85.9) [b]	0.030	0.030	0.13	0.67
**PT (sec)**	8.07 (7.50-8.68)	8.95 (8.43-9.50)	8.77 (8.22-9.36)	0.08	N/A	N/A	N/A
**APTT (sec)**	14.5 [13.3-16.4]	15.5 [14.6-17.5]	16.6 [14.4 – 19.15]	0.058	N/A	N/A	N/A
**vWF (%)**	111.4 (90.4-132.4)	123.9 (106.5-141.3)	112.8 (94.0-131.6)	0.58	N/A	N/A	N/A
