# Supplementary material for: Allergy, inflammation, hepatopathy and coagulation biomarkers in dogs with suspected anaphylaxis due to insect envenomation
Source: Front Vet Sci. 2022 Aug 8;9:875339. doi: 10.3389/fvets.2022.875339 (PMC9393546; doi:10.3389/fvets.2022.875339)
Supplement: Supplementary file 2 [file Data_Sheet_2.PDF]

Supplementary Table 2: Median and Q1 and Q3 for dogs of the entire critical illness group and the critical illness group less the dogs administered bolus fluid therapy prior to sample collection. The critical illness sample size was 30 for all biomarkers except AST, ALT, ALP, bilirubin, CRP and hyaluronan which each had a sample size of 28. The critical illness group without prior bolus fluid administration sample size was 20 for all biomarkers except AST, ALT, ALP, bilirubin, CRP and hyaluronan which each had a sample size of 19. Abbreviations: CRP, C reactive protein; IL, interleukin; CCL2, C-C Motif Chemokine Ligand-2; CXCL8, C-X-C Motif Chemokine Ligand 8; KC, Keratinocyte-derived chemokine; PC, activated protein C; vWF, von Willebrand factor; PT, prothrombin time; APTT, Activated partial thromboplastin time; AT, Antithrombin; AST, Aspartate aminotransferase; ALT, Alanine aminotransferase; ALP, Alkaline phosphatase; MCT, Mast cell tryptase.

	All critical illness group			Critical illness dogs not administered bolus fluid therapy		
**Analyte**	**Median**	**Q1**	**Q3**	**Median**	**Q1**	**Q3**
**Histamine (ng/mL)**	6.87	4.37	8.73	6.87	4.64	8.51
**MCT (ng/mL)**	16.86	10.60	18.96	12.20	8.97	18.02
**IL6 (pg/mL)**	229.48	61.51	548.63	214.98	37.88	581.43
**CXCL8 (pg/mL)**	614.57	212.30	1554.29	697.33	287.31	1881.87
**KC (pg/mL)**	223.94	81.93	506.01	303.07	91.88	773.03
**IL10 (pg/mL)**	23.46	8.63	72.99	31.02	8.63	104.77
**IL18 (pg/mL)**	23.26	8.63	276.57	20.98	8.63	336.21
**CCL2 (pg/mL)**	520.84	234.57	794.89	520.84	239.83	821.76
**CRP (mg/L)**	61.40	14.80	174.95	73.20	42.20	179.80
**Hyaluronan (ng/mL)**	36.16	21.68	55.16	29.86	18.85	54.85
**AST (U/L)**	48.55	36.20	108.10	37.90	31.90	51.60
**ALT (U/L)**	43.95	29.20	141.05	35.30	26.80	58.70
**ALP (U/L)**	92.40	63.90	157.45	87.95	61.10	135.50
**Bilirubin (umol/L)**	1.65	0.45	2.80	1.75	0.70	2.80
**Fibrinogen (g/L)**	3.28	1.62	4.98	3.70	2.21	5.18
**AT (%)**	78.96	71.31	98.20	80.15	73.76	98.23
**PC (%)**	85.21	65.80	111.39	87.26	76.04	110.06
**PT (sec)**	8.65	8.00	9.60	8.65	8.10	9.65
**APTT (sec)**	15.50	14.60	17.50	16.30	14.80	20.80
**vWF (%)**	126.84	66.60	171.68	146.76	65.62	182.70
